# “Extremely cheap and extremely potent”: Fentanyl Knowledge and Risk Practices AmongYoung People in Oregon Who Have Used Non-Prescription Pills

**DOI:** 10.21203/rs.3.rs-8534564/v1

**Published:** 2026-02-13

**Authors:** Danielle Good, Sarah S. Shin, Kyn Kappesser, Erin Stack, Carson Deahl, Courtney Fultineer, Judith M. Leahy, P. Todd Korthuis

**Affiliations:** Comagine Health; Comagine Health; Comagine Health; Comagine Health; Comagine Health; Oregon Health Authority, Injury and Violence Prevention Program, Public Health Division; Oregon Health Authority, Behavioral Health Division; Department of Medicine, Division of General Internal Medicine, Section of Addiction Medicine, Oregon Health & Science University

**Keywords:** fentanyl, counterfeit pills, youth, young people risk reduction, overdose prevention

## Abstract

**Background:**

Overdose deaths involving illicitly manufactured fentanyl have risen sharply, particularly among young people, who are increasing exposed to counterfeit pills. While much is known about adult populations, little research explores how young people perceive and respond to the risks of fentanyl.

**Methods:**

From October 2023 to March 2024, we conducted semi-structured interviews with 21 young people in Oregon (ages 15 to 25) who had reported lifetime use of non-prescription pills. Interviews explored knowledge, experiences, risk reduction practices, and trusted information sources related to fentanyl and counterfeit pills. Transcripts were analyzed using thematic analysis.

**Results:**

Participants demonstrated broad awareness of the dangers of fentanyl and counterfeit pills and described fentanyl as potent, lethal, and increasingly prevalent. Knowledge varied, however, with some holding misconceptions (e.g., risk of overdose from touch). Participants reported practicing risk reduction strategies, including avoiding pills of uncertain sources, using fentanyl test strips, carrying naloxone, and monitoring peers’ safety. Barriers such as limited access to resources, inconsistent availability of drug checking tools, and stigma constrained their ability to reduce risk. Participants described strong peer-to-peer networks that emphasized safety and mutual support. Finally, participants expressed a desire for accurate, fact-based, nonjudgmental education about fentanyl and drug use. They emphasized the importance of schools, parents, and public health organizations providing open dialogue, risk reduction information, and accessible supports such as mental health services, hotlines, and treatment.

**Conclusion:**

Young people in Oregon who have used non-prescription pills are aware of fentanyl’s presence in the drug supply and are motivated to stay safe but encounter barriers that limit their ability to consistently practice risk reduction. Tailored, age-specific interventions that build on existing peer-to-peer networks and expand access to risk reduction resources—including naloxone, fentanyl test strips, and fact-based education—are urgently needed to reduce overdose risk and support young people’s wellbeing.

## 1. Background

Drug overdose deaths involving illicitly manufactured fentanyl have risen to historically high levels across the United States, driven in part by the growing presence of counterfeit pills that mimic prescription medications such as oxycodone, benzodiazepines, and other pharmaceuticals. Over the past several years, overdose mortality among young people ages 15 to 25 has escalated nationally and in Oregon, raising serious public health concerns. While knowledge, attitudes, and beliefs related to fentanyl have been documented among adult populations, comparatively less is known about how young people are navigating an increasingly toxic and unpredictable drug supply.

Nationally, overdose deaths among individuals ages 15–24 increased by approximately 49% between 2019 and 2020, marking one of the largest year-over-year increases observed for this age group (Hedegaard et al., 2021). Although overdose rates have stabilized in subsequent years, overdose mortality among adolescents and young adults remained elevated through 2023 (Miech, 2023). Importantly, this rise in fatalities has not been accompanied by comparable increases in substance use prevalence among young people during the same period (Substance Abuse and Mental Health Services Administration, 2021). Instead, evidence points to changes in the drug supply—including the presence of synthetic opioids such as fentanyl that are manufactured into counterfeit pills, mimicking prescription opioids, benzodiazepines, and other medications. These pills pose particular dangers due to their high potency, unknown or variable composition, and ease of administration (e.g., oral or intranasal rather than injection). Young people aged 15 to 25 bear disproportionate risk when exposed to counterfeit pills containing fentanyl (Daniulaityte et al., 2022, Tanz et al., 2024, Glidden et al., 2024). Younger, opioid naïve individuals are at high risk of accidental overdose because they do not have a tolerance for opioids and may not have access to naloxone (Frank et al., 2015, Goldman-Hasbun et al., 2017). Research highlights the critical role of risk reduction strategies in mitigating overdose risks and promoting safer drug use practices among vulnerable populations (Alper et al., 2024, Friedman et al., 2022, Kimmel et al., 2021). Understanding these trends and interventions is crucial in designing targeted approaches to combat the opioid crisis effectively and protect the health and well-being of young people.

Oregon reflects these national patterns. State surveillance data show that overdose deaths rose sharply between 2021 and 2023, reaching historically high levels, with adolescents and young adults experiencing similar trajectories. Although provisional data indicate a decline in overdose deaths in 2024, mortality remains substantially higher than in earlier years, underscoring that Oregon continues to face a sustained overdose crisis that warrants continued attention, resources, and youth-specific prevention and response efforts (Oregon Health Authority, 2025, Oregon Health Authority, 2022).

We collected responses from young people in Oregon about awareness of fentanyl and non-prescribed pills. We heard from participants about their knowledge of fentanyl, risk reduction strategies, and overdose symptoms and response. Additionally, participants shared their insights into their trusted sources for seeking treatment and obtaining accurate and reliable information about fentanyl. A better understanding of young peoples’ perceptions is needed to effectively develop tailored educational campaigns, interventions, and age-specific services. These interview findings offer in-depth insights into young people’s perceptions of and experiences with fentanyl and counterfeit pills, attitudes and behaviors surrounding risk reduction, and suggestions to better support young people.

## 2. Methods

We implemented an explanatory sequential design study to investigate young people’s knowledge, experiences, and attitudes of fentanyl, counterfeit pills, and risk reduction. The two parts of our study included 1) a cross-sectional online survey (quantitative)(Stack et al., Under Review) and 2) follow-up interviews (qualitative). We developed the survey and interview guide through iterative discussions with the study team, community organization staff, youth treatment providers, and two young adults. We solicited feedback from survey respondents who reported any lifetime non-prescription pill use by asking “What do you think we should ask other young people about?”. We reviewed and analyzed these suggestions to inform the development of the interview guide. We also shared survey findings with community partners and collaborators, who identified high-priority topics. The interview guide included questions to better understand experiences and concerns regarding fentanyl and counterfeit pills knowledge and exposure risk, risk reduction knowledge and behaviors, overdose and response, trusted sources for seeking help, and suggestions to better support young people. We conducted in-person and phone interviews. The study was approved by the Oregon Health and Science University (OHSU) Institutional Review Board (IRB# 17233) and granted a Federal Certificate of Confidentiality.

### 2.1 Participants

We recruited participants (N = 21) from October 13, 2023, to March 6, 2024. To be eligible to participate, participants needed to: 1) be aged 15 to 25 years old, 2) reside in Oregon, and 3) report any lifetime non-prescription pill use (i.e., use of pills not prescribed to them by a doctor or bought from a store).

### 2.2 Recruitment

We recruited participants using three non-probability sampling methods: 1) sequential sampling; 2) snowball sampling; and 3) convenience sampling. The first phase of sequential sampling involved contacting survey respondents who reported any lifetime non-prescription pill use and provided their contact information in a separate, unconnected form for a follow-up interview. Once the initial contact list was exhausted, we employed snowball sampling, asking interview participants to share information about the study with peers who may be interested. Data collectors (SS, KK) contacted participants via phone calls, texts, and emails to confirm their continued interest in participating in a phone interview. Finally, we collaborated with youth-serving organizations and conducted on-site recruitment during service hours. For on-site recruitment, study staff followed an IRB-approved recruitment script to share information about the study.

## 3. Procedures

Data collectors screened interested participants and, if eligible, obtained verbal consent and conducted interviews. Interviews lasted about 60 minutes. Participants received a $40 gift card for their time. All staff have previous experience and training in qualitative data collection and interviewing and working with people who use drugs. Interviewers were trained to follow a protocol if a participant expressed thoughts of suicide, including providing the Suicide Lifeline number, offering to connect the participant to an on-call clinician for crisis counseling, and offering to connect the participant to a peer recovery support specialist for support. Study leadership (ES) regularly reviewed interview audio recordings to provide feedback and ensure interview quality and completeness. The study team met weekly during data collection to discuss interview content. Audio-recorded interviews were transcribed by a professional transcriptionist and uploaded into ATLAS.ti (Web version) for analysis.

### 3.1 Data Analysis

Before analysis, the qualitative analysts participated in a reflexive exercise to identify and discuss any potential biases or preconceptions that could influence data interpretation.

Interviews assessed experiences and attitudes of young people in Oregon who have used non-prescription pills. The COREQ checklist was used, and responses can be found in Appendix A.

We used the interview guide to create the initial codes and an iterative process to refine the codebook and achieve acceptable interrater reliability. Three team members (KK, CD, SS) coded the same transcript and ran a coding comparison query. The first test yielded a low kappa coefficient, so coders reviewed discrepant codes and added clarity to codebook definitions. Coders then coded a second transcript and ran a coding comparison query, achieving a kappa coefficient of > .80, which was deemed sufficient (McHugh, 2012). Coders added additional clarity to codebook definitions and coded the remaining transcripts independently and met regularly to discuss processes and resolve any coding discrepancies. Coded data were then used to construct themes through an iterative inductive process by three team members (KK, SS, ES). Within themes, subthemes were identified, and relationships between and across themes were examined.

## 4. Results

Of the 21 participants, 43% identified as women at the time of the interview and were between the ages of 18–25 (86%), with an average age of 22 years. Nearly half identified as multi-racial (48%) and had completed high school or obtained a GED equivalent (29%). The majority were from urban zip codes (71%) and lived either in a home that they rented or owned (38%) or in their parent or a guardian’s home (24%). About a third of our participants reported housing instability (29%) at the time of their interview. The most commonly reported lifetime non-prescription pill use included ADHD medications (67%), pain pills (62%), and benzodiazepines or MDMA (48%). Past 30-day mental health concerns were prevalent among participants, with 90% experiencing them “Sometimes” (38%), “Most of the time” (24%), or “Always” (29%). Table 1 presents the socio-demographic characteristics of the sample.

We constructed three themes from this data. Young people who have used non-prescription pills (1) have knowledge of and experiences with fentanyl and counterfeit pills; (2) are interested in, aware of, and practicing risk reduction strategies, but face barriers to applying strategies; and (3) are open and interested in receiving support and have recommendations for effective communication and resources. Table 2 presents the themes and supporting quotes.

### 4.1 Young people who have used non-prescription pills have knowledge of and experiences with fentanyl and counterfeit pills.

Participants consistently described fentanyl as dangerous, potent, and often lethal, and they linked its growing presence in the drug supply to rising overdose rates in their communities:

Fentanyl, obviously, everybody knows it’s bad for you, but it’s a lot more potent and dangerous than heroin.

(Participant 5, age 24)

Nowadays, the overdoses go up in the neighborhood. A lot of overdose action starts happening. I noticed that a trend in overdoses, around the time of fentanyl being introduced.

(Participant 15, age 21)

It’s really like the margin of error is so much smaller before you just aren’t okay, and so I think it’s really something that needs to be respected.

(Participant 22, age 22)

I know that fentanyl is extremely cheap and extremely potent. The prevalence is very high because of those two factors. It takes so little to produce the high, and it can be sold for so cheap. It’s just a lot of it is imported, and a lot of people press pills with fentanyl to make them stronger, to make them extremely addictive.

(Participant 2, age 19)

However, participants’ knowledge varied regarding details, such as the types of drugs fentanyl is found in, what it looks like, the difference between pharmaceutical and illicit fentanyl, and what forms (e.g., powder or pill) it comes in. One participant shared,

There’s drug fentanyl, like street fentanyl, and there’s fentanyl that you get at the doctor’s office, and its contamination of pressed pills, fentanyl has started to become a common thing that people are cutting other drugs with, mainly, pressed pills or loose powder just because it is so cheap and readily available and easy to manufacture and relatively colorless and odorless, for the most part.

(Participant 16, age 16)

Other participants spoke more generally about fentanyl, emphasizing its lethal risks without elaborating on its form:

Just that you could OD [overdose] from it, and it could kill you. I don’t really know.

(Participant 7, age 24)

It kills. That’s basically it.

(Participant 13, age 24)

I don’t know much about risks other than “it’s dangerous,” that’s it.

(Participant 3, age 24)

The source of pills also shaped risk perceptions. Pills obtained from family members or peers with prescriptions were generally considered safe, while street purchases or online sources were viewed as unpredictable and dangerous:

I just know that, at this point, it’s just not safe to trust street pills really, at all.

(Participant 2, age 19)

It’s kind of scary because I never bought my own pills. I always got them from somebody who I knew, or who I knew wasn’t going to lace me or do something bad. I really felt pretty confident in what I was getting myself into. I mean, you can’t really be confident with stuff like that, but I was never really worried. I know some people, I’d be worried about them because they’re kind of slower. There’s not as much critical thinking.

(Participant 11, age 16)

I know the ones [pills] prescribed from hospitals and actual doctors, there is no chance that it [fentanyl] would be in there getting it straight from a pharmacy. If it’s off the streets, there’s a 50–50 chance it’s in there.

(Participant 19, age 19)

Participants broadly recognized fentanyl’s dangers, but their perceptions included a mix of accurate knowledge and persistent misconceptions. Two participants who reported less frequent experience and exposure to pills repeated myths common in public discourse, such as overdose from skin contact or incidental inhalation:

It’s strong and it can kill you, even if you have it touch your skin or if you breath in a little bit of particles of it.

(Participant 5, age 24)

It’s deadly, even if you don’t consume fentanyl. Just by either touching it or coming into contact with it, you can die.

(Participant 7, age 24)

### 4.2 Young people who have used non-prescription pills are interested in risk reduction strategies but face barriers to applying strategies.

Some participants chose to avoid pill use altogether or restricted use to prescribed medications or pills from trusted family members. These choices reflected concern about contamination and a desire to maintain control over risk.

The changes me and my friends have made is that we don’t [use pills]. At least me and my closer circle of friends—except for, again, friends who are using ecstasy—but we just don’t do pills.

(Participant 2, age 19)

I don’t use [pills] at all anymore, unless I’m prescribed it. I will wait, honestly, until I can get a doctor’s appointment, and occasionally will still get pain medication from a family member because I know what it is and where it’s coming from, but I will not touch anything else from anyone else.

(Participant 9, age 20)

I’ve just decided to not take pills… Or I will take things that I feel like aren’t usually laced with fet [fentanyl], like tabs.

(Participant 12, age 25)

Participants described using strategies such as carrying naloxone, using fentanyl test strips, avoiding using alone, and taking small “tester shots.” Some also adopted technology-based practices, like sharing their phone location or monitoring vital signs with smartwatches.

Narcan and fentanyl test strips—even more recently xylazine test strips—have become fuckin’ staples for me and all of my homies.

(Participant 16, age 16)

I’m a lot more serious about doing tester shots.

(Participant 22, age 22)

Have a Narcan with you, that’s really what I know. Let someone know what you’re doing or at least be around someone else and Narcan.

(Participant 9, age 20)

I started carrying Narcan because I realized how many people were overdosing, and how big of a problem it is where I live, like just how common it is where I live.

(Participant 16, age 16)

I carry Narcan, test strips, and I always carry food and water on me at all times, so bread, water, and you need to carry a big ass thing of bread and water.

(Participant 17, age 17)

Mutual support emerged as a strong protective factor. Participants described sharing risk reduction information, looking out for their peers, and ensuring that no one used substances alone.

They have a group chat. It’s like if anyone wants to go on some sort of trip or whatever, they’ll just text the group chat and be like: ‘Hey, this is where I am. This is what I’m doing. I will check back in with you guys at this time.’ If they don’t, then people go check on them physically or calling or whatever.

(Participant 3, age 24)

Well, first of all, I tell them, ‘Don’t touch it,’ but if they do, just make sure someone knows; make sure someone’s with you who isn’t using.

(Participant 9, age 20)

Yet participants also highlighted barriers to consistent risk reduction, including uneven access and institutional hurdles:

If you are a social person, it is extremely easy to find [Narcan]. I think the main barrier is people being too messed up to even use the harm reduction in the first place.”

(Participant 2, age 19)

The people who are there for the support program for people who use, you have to find them… In order to get it [Narcan], you have to jump through all these loops, and that doesn’t make any sense to me.”

(Participant 12, age 25)

### 4.3 Young people who have used non-prescription pills are open and interested in receiving support and have recommendations for effective communication and resources.

Participants expressed a strong desire to learn more information about fentanyl and risk reduction, emphasizing the importance of receiving messaging in practical, fact-based, and nonjudgmental ways. Participants identified trusted messengers and channels for fentanyl education and messaging through health care providers, schools, parents, and public health agencies, provided communication was stigma-free and accessible:

Downtown billboards, I know for me, are a big thing… flyers or posters or something. I think keeping the dissemination of information physical would help information to reach the community that need it most.

(Participant 3, age 24)

I honestly feel everyone’s job should have a flyer or something up about it… I think the community pages should be posting more about it. I think the news should be covering it more. Just more visibility on the topic.

(Participant 9, age 20)

Through programs like this [organizations serving unhoused youth]… I think homeless youth struggle the most with getting fentanyl test strips or whatever.

(Participant 13, age 24)

Participants highlighted how supportive, non-stigmatizing conversations and communications with trusted messengers could inform behavior,

I would have liked my parents to tell me, ‘Oh yeah, we don’t really want you to do drugs or take any pills or anything, but if you ever feel the need to, please do it at home. We’ll make sure to get some test kits before. We want you to be in a safe atmosphere.’

(Participant 7, age 24)

If there was a way to have a non-judgmental, in schools in terms of speaking, where your parents wouldn’t know… Instead of faulting that person for doing them, we should be looking at how can we help this person get better? [Creating] an environment with that mindset would probably help a lot with making people more comfortable about seeking help.

(Participant 20, age 22)

Participants also described the types of resources and supports they wanted greater access and awareness to, including hotlines, naloxone, test strips, and mental health services:

Having signs or posters or whatever—just highly visible hotlines to call… other hotlines to people with Narcan or non-profit foundations, stuff like that.

(Participant 2, age 19)

For a homeless person, finding Narcan and test strips is really fuckin’ hard, but [eventually] I got a resupply.

(Participant 17, age 17)

I wish I would have known of more mental health resources, and I wish I would have had more information of certain drugs or side effects or fentanyl in general.

(Participant 7, age 24)

Finally, participants described how stigma and the absence of trust with supportive adults hindered willingness to seek help and pursue conversations surrounding pill use.

I’ve never talked to my parent… When I did mention that I was abusing pills, specifically, I was kind of laughed at. No. Anything that I learned about it was on my own.

(Participant 21, age 22)

Not really, just because no one really seems to care except for you all. You all are the first people who really asked me about any sort of anything other than where to get it.

(Participant 14, age 25)

## 5. Discussion

This study adds to a growing body of research examining how young people perceive and respond to the risks of fentanyl and counterfeit pills. Consistent with recent epidemiological reports documenting sharp increases in adolescent overdose deaths linked to illicit fentanyl (Friedman et al., 2022, Banta-Green, Williams, 2021, Spencer, 2024), our participants demonstrated broad awareness of fentanyl’s dangers and its growing presence in their communities. Participants also engaged in source-based risk calculations—treating pills from family or peers with prescriptions as relatively safe while viewing “street pills” or online purchases as unpredictable and dangerous. This nuanced perception mirrors findings from qualitative research on counterfeit pill use (Daniulaityte et al., 2022, Arya et al., 2022) and studies documenting the spread of fentanyl through pressed pills and counterfeit oxycodone (Friedman, Ciccarone, 2025, O’Donnell, 2023). Our findings extend the existing literature by demonstrating how young people perceive and respond to source reliability as a crucial factor in their decision-making.

Beyond awareness, participants described active risk reduction practices. Many reported avoiding non-prescription pills and using alone, carrying naloxone, using fentanyl test strips, and employing collective safety measures such as group chats, location sharing, or designating a sober friend. These practices resonate with prior studies highlighting young adults’ willingness to use fentanyl test strips (Krieger et al., 2018a, Krieger et al., 2018b) and drug checking among festival-goers (Day et al., 2018), as well as broader frameworks that emphasize developmentally appropriate risk reduction for young people (Kimmel et al., 2021, Winer et al., 2022). At the same time, participants highlighted barriers including inconsistent access to naloxone and drug checking tools, institutional restrictions, and stigma—echoing findings from work on barriers to naloxone uptake (Bennett et al., 2020) and service delivery challenges for young people (Marchand et al., 2023). These results suggest that young people are motivated to stay safe, but structural and social constraints shape their ability to apply risk reduction consistently.

Young people emphasized looking out for one another, ensuring no one used alone, and sharing resources. This collective orientation stands in contrast to dominant narratives that pathologize youth drug use (Collins et al., 2018, Farrugia, 2014) and aligns with work documenting the centrality of peer support in shaping risk and resilience among marginalized young people (Boyd et al., 2017, Stowe et al., 2022). Interventions that strengthen rather than undermine these peer systems—such as peer-led naloxone distribution, existing risk reduction tools like the *Never Use Alone* hotline, or digital tools that formalize safety check-ins—may therefore resonate more strongly than purely top-down approaches.

Participants also described clear preferences for messaging and support. They consistently rejected fear-based narratives and called for information that was factual, nonjudgmental, and age-appropriate. Schools, parents, and public health agencies were identified as trusted messengers, provided communication was open and stigma-free. This aligns with evaluations of school-based curricula like Safety First, which improved risk reduction knowledge and behaviors (Fischer, 2022), but also with critiques of punitive school climates that reproduce inequities and deter help-seeking (Duarte et al., 2023). Similar to research on health messaging in social media contexts (Lee et al., 2024), our findings suggest that who delivers the message and how it is framed are critical. In practice, participants wanted visible, accessible resources—such as posters, hotlines, and easy and discrete access to naloxone and test strips—rather than abstract warnings.

Finally, our study underscores the persistent gap between risk awareness and treatment access. Even after overdose, young people rarely receive evidence-based treatment (Alinsky et al., 2020) and residential programs in the United States underutilize medications for opioid use disorder (King et al., 2023), despite clinical consensus supporting them for this age group (Society for Adolescent, Medicine, 2021). In Oregon, these challenges are exacerbated by substantial shortages in youth-specific treatment and recovery services, including limited availability of developmentally appropriate care, uneven geographic access, and a statewide behavioral health system operating far below estimated need (Oregon Health Authority, 2023, Lenahan et al., 2022). Participants’ experiences highlight the urgency of integrating risk reduction with age-appropriate treatment and recovery pathways. Commentaries note that the landscape of adolescent substance use is rapidly changing (Bell, Hadland, 2024), and evidence from Oregon and nationally indicates that system inaction has left young people vulnerable to fentanyl’s spread (Green, 2023, Oregon Health Authority, 2024). Bridging this gap requires implementation of age-tailored risk reduction strategies alongside accessible treatment, ideally with formal mechanisms for elevating young peoples’ voices in program design (Office of Juvenile Justice and Delinquency Prevention, 2023).

## 6. Limitations

Most of our sample were from between the ages of 18 and 25 (86%) and resided in urban areas (71%), findings cannot be applied to the knowledge, behaviors, and recommendations for all young people, more research is needed to better understand experiences and perceptions of youth (ages 15 to 17) in rural areas.

## 7. Conclusion

Young people in Oregon who have used non-prescription pills are navigating an increasingly unpredictable and dangerous drug supply with mixed awareness and preparedness. Participants varied in their depth of knowledge about fentanyl, but many described adapting their behaviors in ways that reflect risk reduction values—testing drugs when possible, avoiding use alone, ensuring peers were present or responsive, and carrying naloxone. Ultimately, protecting young people from fentanyl-related harms requires acknowledging the social realities in which they use drugs and the peer systems they rely on for safety. Interventions that strengthen these networks, reduce barriers to risk reduction supplies, and promote open, stigma-free dialogue about substance use will be most likely to resonate with young people and meaningfully reduce overdose risk.

## Figures and Tables

**Table 1:** Participant Socio-Demographic Characteristics

Age	N = 21	Percent	Mean
15–17	3	14%	22.2
18–25	18	86%	
Gender	N = 21	Percent	
Woman	9	43%	
Man	7	33%	
Non-binary	2	10%	
I am not sure of my gender identity	1	5%	
Trans Male/Non-binary	1	5%	
Man/Non-binary	1	5%	
Race and Ethnicity	N = 21		
Black/African American	1	5%	
Hispanic or Latinx	2	10%	
White	8	38%	
Multiracial	10	48%	
Multiracial	n = 10		
American Indian/Native America + White	2	10%	
American Indian/Native America + White + Black or African American	1	5%	
American Indian/Native America + Hispanic or Latino/Latina/Latinx	1	5%	
Hispanic or Latino/Latina/Latinx + Filipino/a/x	1	5%	
Filipino/a/x + White	1	5%	
Hispanic or Latino/Latina/Latinx + White	3	14%	
Korean + White	1	5%	
Education	N = 21	Percent	
Bachelor’s degree	2	10%	
Associate degree	1	5%	
Some colleges	5	24%	
High school graduate or GED	6	29%	
Some high school	4	19%	
Less than high school	3	14%	
Housing	N = 21	Percent	
In a home that I rent or own	8	38%	
In my parent’s or guardian’s home	5	24%	
In a shelter or emergency housing	3	14%	
In the home of a friend, family member, or other person	2	10%	
Transitional housing	2	10%	
In a car, park, campground, or other public place	1	5%	
Sexual Orientation	N = 21	Percent	
Queer	4	19%	
Bisexual	5	24%	
Gay	1	5%	
Lesbian	1	5%	
Heterosexual	7	33%	
Pansexual	3	14%	
Food Insecurity (Past 30-days)	N = 21	Percent	
Never	5	24%	
Rarely	9	43%	
Sometimes	4	19%	
Most of the time	3	14%	
Always	0	0%	
Mental Health Concerns (Past 30-days)	N = 21	Percent	
Never	0	0%	
Rarely	2	10%	
Sometimes	8	38%	
Most of the time	5	24%	
Always	6	29%	
Lifetime non-prescription pill use	N = 21	Percent	
ADHD medications	15	71%	
Pain medications	12	57%	
Benzodiazepines	9	43%	
MDMA	9	43%	
Fentanyl pills	4	19%	
Antidepressants	3	14%	
Gabapentin	3	14%	
Buprenorphine pills	1	5%	
Geographic	N = 21	Percent	
Urban	15	71%	
Rural	6	29%	

**Table 2:** Themes and supporting quotes

Theme	Supporting Quotes
*Young people who have used non-prescription pills have knowledge of and experiences with fentanyl and counterfeit pills*.	Fentanyl, obviously, everybody knows it’s bad for you, but it’s a lot more potent and dangerous than heroin. (Participant 5, age 24) Nowadays, the overdoses go up in the neighborhood, heavy. A lot of overdose action starts happening. I noticed that a trend in overdoses, around the time of fentanyl being introduced. (Participant 15, age 21) It’s really like the margin of error is so much smaller before you just aren’t okay, and so I think it’s really something that needs to be respected. (Participant 22, age 22) I know that fentanyl is extremely cheap and extremely potent. The prevalence is very high because of those two factors. It takes so little to produce the high, and it can be sold for so cheap. It’s just a lot of it is imported, and a lot of people press pills with fentanyl to make them stronger, to make them extremely addictive. (Participant 2, age 19) There’s drug fentanyl, like street fentanyl, and there’s fentanyl that you get at the doctor’s office, and its contamination of pressed pills, fentanyl has started to become a common thing that people are cutting other drugs with, mainly, pressed pills or loose powder just because it is so cheap and readily available and easy to manufacture and relatively colorless and odorless, for the most part. (Participant 16, age 16) Just that you could OD [overdose] from it, and it could kill you. I don’t really know. (Participant 7, age 24) It kills. That’s basically it. (Participant 13, age 24) I don’t know much about risks other than “it’s dangerous,” that’s it. (Participant 3, age24) I just know that, at this point, it’s just not safe to trust street pills really, at all. (Participant 2, age 19) It’s kind of scary because I never bought my own pills. I always got them from somebody who I knew, or who I knew wasn’t going to lace me or do something bad. I really felt pretty confident in what I was getting myself into. I mean, you can’t really be confident with stuff like that, but I was never really worried. I know some people, I’d be worried about them because they’re kind of slower. There’s not as much critical thinking. (Participant 11, age 16) I know the ones [pills] prescribed from hospitals and actual doctors, there is no chance that it [fentanyl] would be in there getting it straight from a pharmacy. If it’s off the streets, there’s a 50–50 chance it’s in there. (Participant 19, age 19) It’s strong and it can kill you, even if you have it touch your skin or if you breath in a little bit of particles of it. (Participant 5, age 24) It’s deadly, even if you don’t consume fentanyl. Just by either touching it or coming into contact with it, you can die. (Participant 7, age 24)
*Young people who have used non-prescription pills are interested in risk reduction strategies but face barriers to applying strategies*.	The changes me and my friends have made is that we don’t [use pills]. At least me and my closer circle of friends—except for, again, friends who are using ecstasy—but we just don’t do pills. (Participant 2, age 19) I don’t use [pills] at all anymore, unless I’m prescribed it. I will wait, honestly, until I can get a doctor’s appointment, and occasionally will still get pain medication from a family member because I know what it is and where it’s coming from, but I will not touch anything else from anyone else. (Participant 9, age 20) I’ve just decided to not take pills… Or I will take things that I feel like aren’t usually laced with fet [fentanyl], like tabs. (Participant 12, age 25) Narcan and fentanyl test strips—even more recently xylazine test strips—have become fuckin’ staples for me and all of my homies. (Participant 16, age 16) I’m a lot more serious about doing tester shots. (Participant 22, age 22) Have a Narcan with you, that’s really what I know. Let someone know what you’re doing or at least be around someone else and Narcan. (Participant 9, age 20) I started carrying Narcan because I realized how many people were overdosing on this, and how big of a problem it is where I live, like just how common it is where I live. (Participant 16, age 16) I carry Narcan, test strips, and I always carry food and water on me at all times, so bread, water, and you need to carry a big ass thing of bread and water. (Participant 17, age 17) They have a group chat. It’s like if anyone wants to go on some sort of trip or whatever, they’ll just text the group chat and be like: ‘Hey, this is where I am. This is what I’m doing. I will check back in with you guys at this time.’ If they don’t, then people go check on them physically or calling or whatever. (Participant 3, age 24) Well, first of all, I tell them, ‘Don’t touch it,’ but if they do, just make sure someone knows; make sure someone’s with you who isn’t using. (Participant 9, age 20) If you are a social person, it is extremely easy to find [Narcan]. I think the main barrier is people being too messed up to even use the harm reduction in the first place.” (Participant 2, age 19) The people who are there for the support program for people who use, you have to find them… In order to get it [Narcan], you have to jump through all these loops, and that doesn’t make any sense to me.” (Participant 12, age 25)
*Young people who have used non-prescription pills are open and interested in receiving support and have recommendations for effective communication and resources*	Downtown billboards, I know for me, are a big thing… flyers or posters or something. I think keeping the dissemination of information physical would help information to reach the community that need it most. (Participant 3, age 24) I honestly feel everyone’s job should have a flyer or something up about it… I think the community pages should be posting more about it. I think the news should be covering it more. Just more visibility on the topic. (Participant 9, age 20) Through programs like this [organizations serving unhoused youth]… I think homeless youth struggle the most with getting fentanyl test strips or whatever. (Participant 13, age 24) I would have liked my parents to tell me, ‘Oh yeah, we don’t really want you to do drugs or take any pills or anything, but if you ever feel the need to, please do it at home. We’ll make sure to get some test kits before. We want you to be in a safe atmosphere.’” (Participant 7, age 24) If there was a way to have a non-judgmental, in schools in terms of speaking, where your parents wouldn’t know… Instead of faulting that person for doing them, we should be looking at how can we help this person get better? [Creating] an environment with that mindset would probably help a lot with making people more comfortable about seeking help. (Participant 20, age 22) Having signs or posters or whatever—just highly visible hotlines to call… other hotlines to people with Narcan or non-profit foundations, stuff like that. (Participant 2, age 19) For a homeless person, finding Narcan and test strips is really fuckin’ hard, but [eventually] I got a resupply. (Participant 17, age 17) I wish I would have known of more mental health resources, and I wish I would have had more information of certain drugs or side effects or fentanyl in general. (Participant 7, age 24) I’ve never talked to my parent… When I did mention that I was abusing pills, specifically, I was kind of laughed at. No. Anything that I learned about it was on my own. (Participant 21, age 22) Not really, just because no one really seems to care except for you all. You all are the first people who really asked me about any sort of anything other than where to get it. (Participant 14, age 25)

## Data Availability

The datasets generated and analyzed during the current study are not publicly available for reasons of confidentiality. The qualitative data collected in this study could be used to identify participants and is therefore only available to the study team. It is protected by a Federal Certificate of Confidentiality.
